# Grazing reshapes plant nitrogen use trade‐off between soil uptake and internal remobilization

**DOI:** 10.1111/tpj.71168

**Published:** 2026-10-12

**Authors:** Lin Wu, Kexin Li, Ziyuan Ma, Hao Wang, Yanlong Li, Yadong Wang, Pengfei Xing, Zhicheng Jin, Tongrui Zhang, Frank Yonghong Li

**Affiliations:** ^1^ Ministry of Education Key Laboratory of Ecology and Resource Use of the Mongolian Plateau, Inner Mongolia Key Laboratory of Grassland Ecology, School of Ecology and Environment Inner Mongolia University 235 West University Road Hohhot 010021 China; ^2^ Collaborative Innovation Center for Grassland Ecological Security, Ministry of Education of China Inner Mongolia University Hohhot 010021 China; ^3^ Observation and Research Station for Steppe Ecosystems of the Ministry of Education of China Xilingol Inner Mongolia China; ^4^ Key Laboratory for Model Innovation in Forage Production Efficiency, Ministry of Agriculture and Rural Affairs, Institute of Grassland Research Chinese Academy of Agricultural Sciences Hohhot 010010 China

**Keywords:** *Leymus chinensis*, grassland ecosystem, ^15^N isotope labelling, plant N use strategy, root N remobilization, soil N uptake, seasonal grazing

## Abstract

Perennial plants rely on two key ecological strategies to supply nitrogen (N) for photosynthetic tissue construction: root N remobilization (internal cycling) and soil N uptake. How plants trade‐off these strategies determines the economy of N use and is likely context‐dependent. Yet, it is rarely quantified *in situ* across growth stages and under varying management, limiting the mechanistic understanding of how plants rebalance N‐use trade‐offs to adapt to grazing disturbance. To address this gap, we conducted an *in situ* experiment using ^15^N isotope labelling techniques in a semiarid grassland to quantify the contributions of these two strategies at two plant growth stages under nil, summer or autumn grazing. We found that the plant relied more on soil N uptake (58%) during the regreen stage, but more on N remobilization (57%) during the rapid‐growth stage. Grazing, especially in autumn, significantly leaned N use trade‐off toward conservative N remobilization. The shift in plant N use strategies was driven primarily by plant growth demand and soil N supply, but not root N stock. Plant N use trade‐off leaned toward internal cycling when source traits were below or sink traits were above the thresholds, for example, soil N availability <9.0 mg kg^−1^ or shoot carbon content >41.88%. Our study highlights the importance of the N resource‐sink relations in mediating the trade‐off in plant N use strategies, buffering grazing stress to grassland plants. These findings provide a mechanistic basis for improving ecosystem N cycling modeling and adaptive grazing management.

## Significance Statement

Perennial herbaceous plants acquire N for aboveground growth through two major processes: root N remobilization and soil N uptake. Using an *in situ* ^15^N tracer technique, we show that grazing significantly leaned N use trade‐off from soil N uptake toward root N remobilization, and the trade‐off is driven by plant growth N demand and soil N supply, rather than root N stock. The trade‐off buffers plants from seasonal and grazing‐induced soil N limitation.

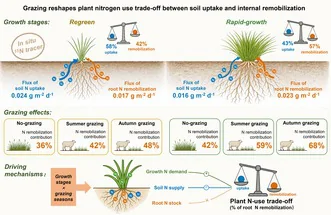

## INTRODUCTION

Perennial herbaceous plants acquire nitrogen (N) for aboveground growth through two major processes: remobilizing N from root storage (internal N cycling) and taking up N from soil (external N cycling) (Thornton & Millard, 1993; Vriet et al., 2014). Root N remobilization is generally a more conservative and economic strategy compared with soil N uptake, as it costs less energy (Brant & Chen, 2015). The trade‐off between these two N acquisition pathways represents the plant economic spectrum of N use ranging from a resource‐acquisitive to conservative strategy (Wang et al., 2024; Zhang et al., 2025). A major challenge in understanding how herbaceous plants balance N use trade‐offs is to quantify them *in situ*, because the underlying strategies are likely contingent on environmental and management context (Liu et al., 2025). Grazing, a common practice in grasslands, may sharply reshape the trade‐offs in plant N use strategies by modifying plant growth demand and soil N supply capacity (Liu et al., 2022; Zhang et al., 2020). Yet, its effects may be stage‐dependent, as plants adjust N use trade‐offs to satisfy changing N requirements through the growing season (Yuan et al., 2007). Given the widespread use of grasslands for grazing, it is essential to quantify how plants adjust N use trade‐offs across growth stages and in response to grazing disturbance for understanding the dynamics of efficient plant N use in grassland ecosystems.

The potential grazing impacts on the plant N use trade‐off have been implied in several defoliation studies. Contributions of N remobilization to herbaceous plant regrown tissues in a short‐term period after defoliation range from 30 to 97% (Wang et al., 2021; Yang et al., 2023), indicating a potentially broad response of the plant N use trade‐off to grazing. Beyond defoliation, grazing‐related changes in environmental conditions may generate indirect effects. Grazing generally leads to soil N loss and a decline in plant root mass and N storage (Cao et al., 2024; Li, Shi, et al., 2025). It likely leads to reductions in the potential of both N remobilization and uptake (Marmagne et al., 2022; Zhang et al., 2023). Conversely, patchy N enrichment in excreta hotspots may enhance both N strategies by strengthening root reserves and soil N supply capacity (Hodge, 2004; Liu et al., 2016). Hence, grazing impacts on N use trade‐offs are expected to be contingent on the relative strength of grazing impacts on two strategies.

Growth season variation in N use trade‐offs has been evidenced in woody plants. Internal cycling supplies plant N demand (contributing to 50–80%) in early growth season (Grelet et al., 2001; Neilsen et al., 1997), but progressively shifts to root uptake later in the season (contributing to 50–70%) (Birkhold & Darnell, 1993; Deng et al., 1989; Millard & Proe, 1992). Herbaceous plants may adjust N‐use strategies more strongly across growth stages than woody plants, potentially because of smaller N storage capacity in belowground tissues including roots, rhizomes, and stem bases (Lipson et al., 1996; Zhang et al., 2025). Existing studies indicate that early growth in herbaceous plants relies more on N remobilization (contributing to 54–82%), whereas information for other periods remains limited (Bausenwein & Millard, 2001; Frak et al., 2002). Notably, most evidence is derived from greenhouse studies (hydroponics or pots), in which the well‐controlled resource supply may introduce systematic bias in N‐use trade‐off estimates. Therefore, there is an urgent need to clarify the relative roles of the two N use strategies *in situ*, to refine our understanding of how plants achieve efficient N use by adjusting this trade‐off.

Understanding the responses of plant N use trade‐off requires disentangling the dominant pathways underpinning the interaction between grazing and growth stage. Plant N acquisition is driven by the interaction of source and sink (Jordan et al., 2012; Lestienne et al., 2006). Source availability, including root storage and soil supply, directly modulates the trade‐off between plant N use strategies (Wang et al., 2021). The cost associated with nutrient acquisition is identified as a critical factor governing root nutrient uptake (Yang et al., 2022); thus, the relative strength of different N sources may potentially determine the trade‐off in plant N use. Plants rely more on conservative internal cycling in N‐deficit habitats (Vitousek & Field, 2001) but also tend to have reduced belowground N storage under N‐deficit conditions, as reported in *Calamagrostis epigejos* (Gloser et al., 2007), *Bistorta bistortoides* (Lipson et al., 1996), and *Lolium perenne* (Ourry et al., 1990). This suggests that the N availability (source) can be a dual control mechanism for the synchronized changes in both N use strategies (Bicharanloo et al., 2022), while the shift in their trade‐off will depend on their relative magnitudes (Wang et al., 2021).

By contrast, the role of plant N demand (sink) has not been considered in the mechanistic framework of plant N use trade‐off (Stocker et al., 2025). Plant growth is fundamentally governed by the tight coupling of carbon (C) assimilation and N utilization, wherein plants regulate their C: N stoichiometry to optimize growth rates (Ågren, 2004; Knecht & Goransson, 2004). The plant leaf economics spectrum demonstrates a universal positive correlation between plant leaf N concentration and photosynthetic capacity (Wright et al., 2004). Therefore, plants with a fast‐growing rate and stronger photosynthetic capacity likely rely more on a resource acquisitive root uptake strategy to fuel growth demand (Wang et al., 2024). The switch in plant N use strategies between root N remobilization and soil N uptake can be regulated by the relations between sink demand and source availability, and there may exist thresholds governing this process. However, no dedicated empirical evidence exists to confirm this sink‐source‐driven framework for plant N use strategies.

We aimed to understand the response of the plant N use trade‐off to grazing across the growing season, and clarify the key mechanistic path. Here, we conducted an *in situ* ^15^N labelling experiment to quantify the trade‐off between root N remobilization and soil N uptake across different plant growth stages in a semiarid grassland that was grazed by sheep in summer (SG) or autumn (AG) *versus* no grazing (NG) for 8 years. We measured the traits representing plant N stock, soil N supply, and growth N demand to assess the relative influence of source versus sink processes on plant N use trade‐off, and for testing the following three hypotheses: (1) Grazing leads plant N use trade‐off leaning toward conservative N remobilization; (2) grazing impacts on plant N use trade‐off are growth stage dependent, with relatively greater reliance on remobilization at early stages but greater reliance on soil N uptake at later stages; and (3) seasonal and grazing‐induced variation in plant N use trade‐off is jointly regulated by N source and sink traits. Validating these hypotheses will provide novel insights into plant internal N cycling and ecosystem N cycling in grazed grasslands and help develop precise nutrient management strategies.

## RESULTS

### Effects of grazing and plant growth stage on the fluxes of root N remobilization and soil N uptake and their trade‐off

Both grazing and plant growth stage had significant effects on the fluxes of root N remobilization and soil N uptake, as well as on the trade‐off between these two N processes. Grazing and plant growth stage also interactively and significantly affect the plant N use trade‐off (Table 1).

**Table 1:** Results of linear mixed models on the effects of grazing and plant growth stage on the fluxes of root N remobilization (Fre), soil N uptake (Fup), and their trade‐off (Cre: contribution of root N remobilization to plant aboveground N accumulation)

Variables	Fre			Fup			Cre		
	df	*F*	*P*	df	*F*	*P*	df	*F*	*P*
Grazing	2	7.90	**0.008**	2	4.74	**0.022**	2	45.59	**<0.001**
Growth stage	1	7.89	**0.013**	1	10.71	**0.004**	1	81.39	**<0.001**
Grazing × Growth stage	2	1.86	0.189	2	1.13	0.346	2	6.69	**0.008**

*F*‐values with associated degrees of freedom (df) and *P*‐values are reported. Sample size is 24. Bold *P*‐values are significant at *P* <0.05.

Compared to no‐grazing, summer and autumn grazing significantly reduced the flux of soil N uptake, whereas autumn grazing significantly enlarged the flux of root N remobilization (Figure 1A). Thus, the contribution of root N remobilization to plant aboveground N accumulation was larger in grazed (50% and 58% under summer and autumn grazing, respectively) than in ungrazed grasslands (39%) (Figure 1C). The flux of soil N uptake was larger, whereas that of root N remobilization was smaller, in the plant regreen stage than the rapid‐growth stage (Figure 1B), which led to a relatively larger contribution of soil N uptake at regreen stage, but a larger contribution of root N remobilization at the rapid‐growth stage (Figure 1D).

**Figure 1:**
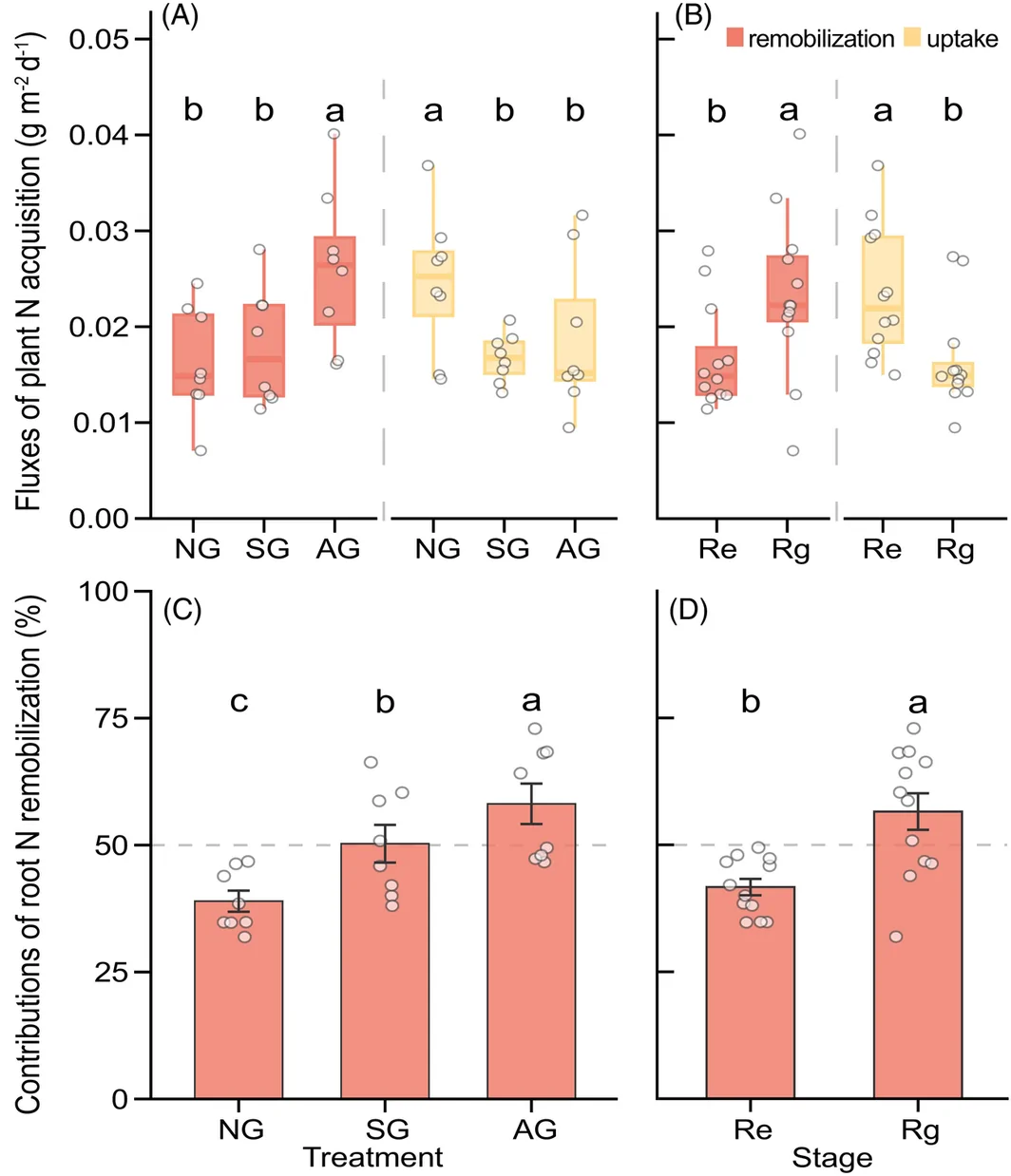
The trade‐off in plant aboveground N use strategies in a semi‐arid grassland in response to three grazing treatments and during two plant growth stages. (A,B) Fluxes of plant aboveground N acquisition through root N remobilization and soil N uptake under three grazing treatments (A) or during two growth stages (B). (C, D) The trade‐off in plant N use strategies (contribution of root N remobilization in N acquisition) under three grazing treatments (C) or during two plant growth stages (D). NG, no‐grazing; SG, summer grazing; AG, autumn grazing; Re, regreen stage; Rg, rapid‐growth stages. Values above 50% (dashed line) indicate dominance of internal N cycling. Box plots show the median (center line) and 25th and 75th percentiles. Column charts show mean ± SE. Different letters indicate significant differences among grazing treatments (A, C) or between growth stages (B, D) (LSD test, *P* < 0.05, *n* = 24).

A significant interaction was detected between grazing and growth stages on the contribution of root N remobilization to plant aboveground N accumulation, but the interaction was not significant on the two N acquisition fluxes (Table 1). Grazing impact on the contribution was consistent across two growth stages, that is, both summer and autumn grazing enhanced the contribution of root N remobilization regardless of growth stage; however, the enhancement was larger under autumn than under summer grazing (Figure 2). The contribution of root N remobilization became larger than that of soil N uptake under grazing at the rapid‐growth stage, while it was always smaller than that of soil N uptake during the plant regreen stage in all cases.

**Figure 2:**
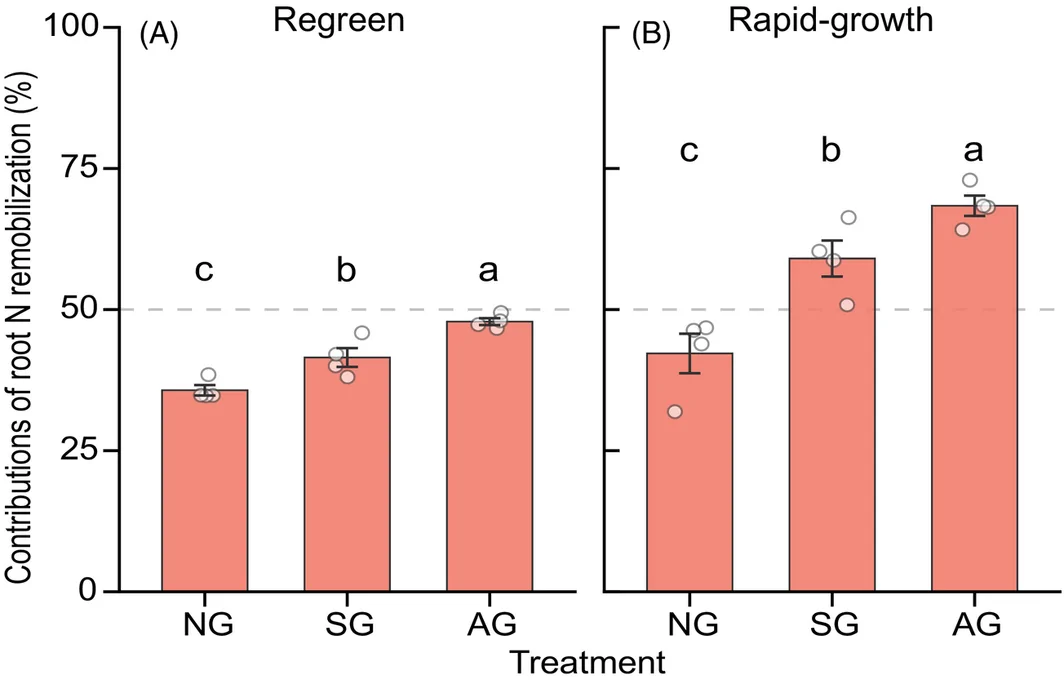
Contributions of root N remobilization to plant aboveground N accumulation under three grazing treatments and during two plant growth stages. (A,B) Contributions of root N remobilization to plant aboveground N accumulation during the regreen stage (A) and the rapid‐growth stage (B). NG, no‐grazing; SG, summer grazing; AG, autumn grazing. Values above 50% (dashed line) indicate dominance of internal N cycling. The column chart with error bars shows mean ± SE. Different letters indicate significant differences among grazing treatments (LSD test, *P* < 0.05, *n* = 12).

### The source and sink factors mediating the fluxes of N remobilization and uptake and their trade‐off

The hierarchical partitioning analysis quantified the roles of plant aboveground growth N demand, root N stock, and soil N supply in driving the variation in plant aboveground N acquisition. The relative importance of predictor variables differed between the two N fluxes and the contribution of root N remobilization. Plant aboveground growth N demand (quantified using the changes in aboveground biomass and shoot C content) explained 76% of the variation in the root N remobilization flux, with shoot C content being the most important predictor (Figure 3A–C). By contrast, soil N supply was the dominant driver of the soil N uptake flux, explaining 60% of its variation, with soil available N as the key predictor (Figure 3D–F). Soil N supply was also the most important factor regulating the trade‐off in plant N use strategies, explaining 52% of its variance, with soil N availability (AN) negatively correlated with the contribution of root N remobilization (Figure 3G–I). Root N stock accounted for little of the variance in either N flux or their trade‐off (Figure 3).

**Figure 3:**
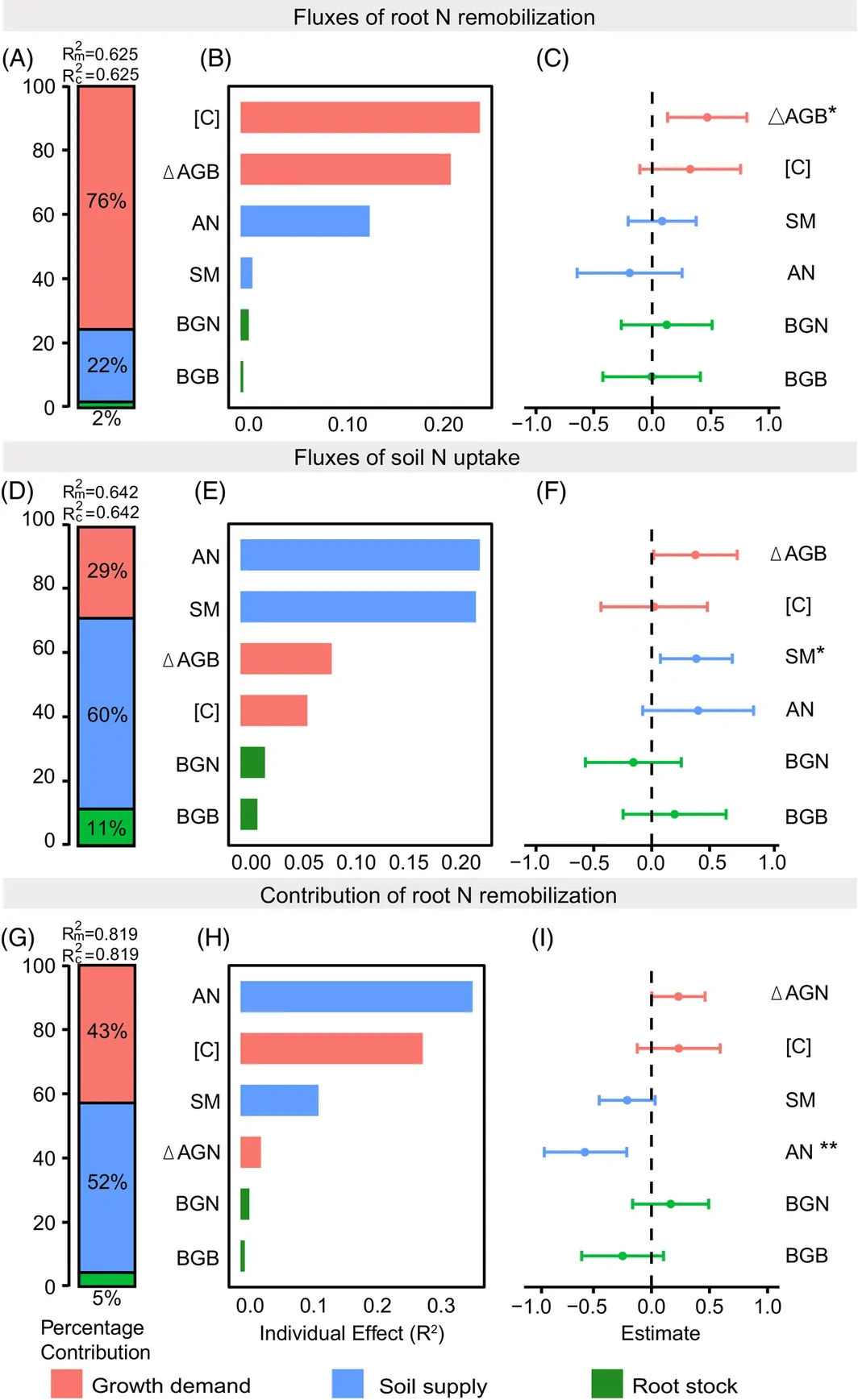
Relative contributions of plant aboveground growth N demand, soil N supply, and root N stock in explaining the variations in plant N use strategies. (A‐C) Relative contributions in explaining the variations in root N remobilization flux. (D‐F) Relative contributions in explaining the variations in soil N uptake flux. (G‐I) Relative contributions in explaining the variations in their trade‐off (contribution of root N remobilization (Cre)). Plant aboveground growth N demand traits were represented by shoot C content ([C]), and changes in plant aboveground biomass (ΔAGB) and nitrogen pool (ΔAGN). Soil N supply traits were represented by soil moisture (SM) and soil N availability (AN). Root N stock traits were represented by plant belowground biomass (BGB) and N pool (BGN). Bar heights represent the relative importance of predictors. Independent explanatory power of each predictor was quantified using the R^2^ value from linear mixed models (A, B, D, E, G, H), with R^2^_m_ and R^2^_c_ representing the marginal and conditional R^2^, respectively. The forest plots (C, F, I) display standardized regression coefficients (points) and their 95% confidence intervals (CIs; horizontal bars) from linear mixed models, with significance marked as *P < 0.05; **P < 0.01; ***P < 0.001. Sample size is 24.

The factors affecting the trade‐off between two plant N use strategies vary with grazing treatments and growth stages. The variance of plant N use trade‐off was dominantly driven by soil N supply in ungrazed grasslands (Figure 4A) but was jointly driven by plant growth demand and soil N supply in grazed grasslands (Figure 4B,C). Soil N availability played a predominant role in ungrazed grasslands, while changes in plant aboveground N pool (ΔAGN), soil moisture (SM), soil N availability (AN), and shoot C content (C) all played important roles in grazed grasslands in driving the variation in plant N use trade‐off. Plant aboveground growth N demand explained most of the variation in the plant N use trade‐off at the regreen stage, with C as the most predominant predictor. In contrast, soil N supply explained most of the variation at the rapid‐growth stage, with AN as the most predominant predictor (Figure 4D,E).

**Figure 4:**
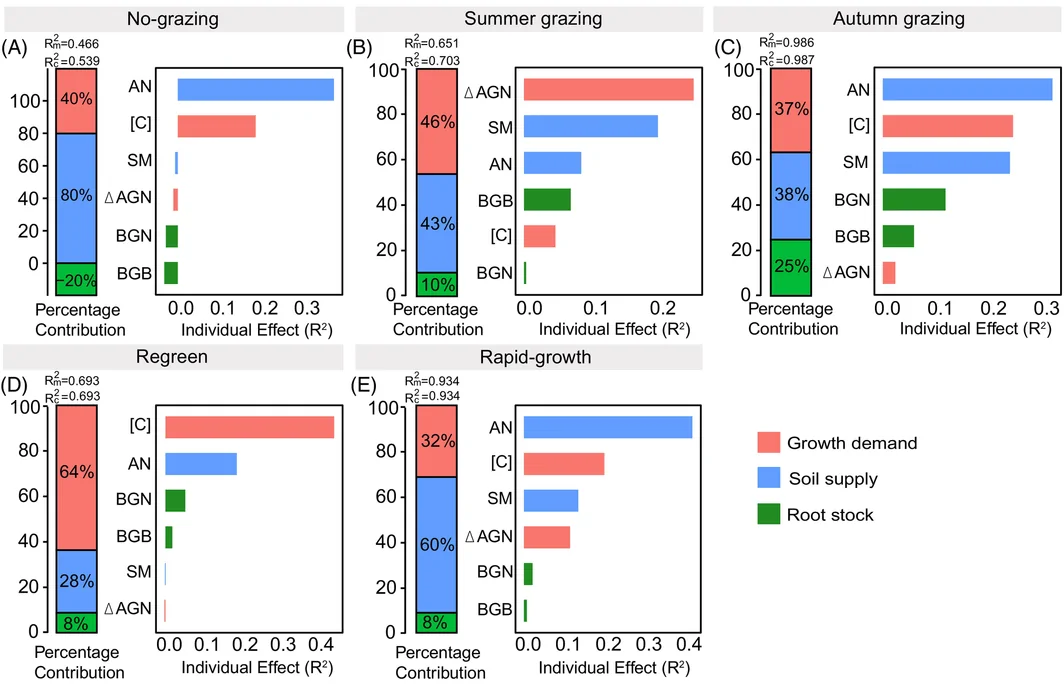
Relative contributions of plant aboveground growth demand, soil N supply, and root N stock in explaining the variations in the trade‐off in plant N use strategies (contribution of root N remobilization in N acquisition). (A‐C) Relative contributions of plant aboveground growth demand (denoted by changes in plant aboveground N pool (ΔAGN) and shoot C content ([C])), soil N supply (denoted by soil N availability (AN) and soil moisture (SM)), and root N stock (denoted by plant belowground biomass (BGB) and plant belowground N pool (BGN)) under three grazing treatments. (D, E) Relative contributions during two plant growth stages. Bar heights represent the relative importance of predictors. The independent explanatory power of each predictor was quantified using the R^2^ value from linear mixed models, with R^2^_m_ and R^2^_c_ representing the marginal and conditional R^2^, respectively. AN, soil N availability; SM, soil moisture; ΔAGN, changes in plant aboveground N pool; [C], shoot C content; BGB, plant belowground biomass; BGN, plant belowground N pool. Sample size is 24.

Plant aboveground growth N demand or soil N supply played key roles in governing the shift in plant N use strategies. In both plant growth stages, summer or autumn grazing increased plant growth N demand traits (shoot C content, Figure 5A,E), but reduced soil N supply traits (soil N availability, soil moisture, Figure 5C,G,I). Correlation analysis showed that shoot C content was positively correlated (Figure 5B,F), while soil N availability and moisture were negatively correlated (Figure 5D,H,J) with the contribution of N remobilization in both regreen and rapid‐growth stages. At the regreen stage, the observed contributions were consistently below 50% across all treatments (Figure 5B,D). By contrast, an evident transition of plant N use trade‐off was detected during the rapid‐growth stage. Plant N use shifted from greater reliance on soil N uptake to greater reliance on root N remobilization when shoot C content increased above approximately 41.88% (Figure 5F), or when soil N availability or moisture decreased below 9.0 mg kg^−1^ and 9.82%, respectively (Figure 5H,J).

**Figure 5:**
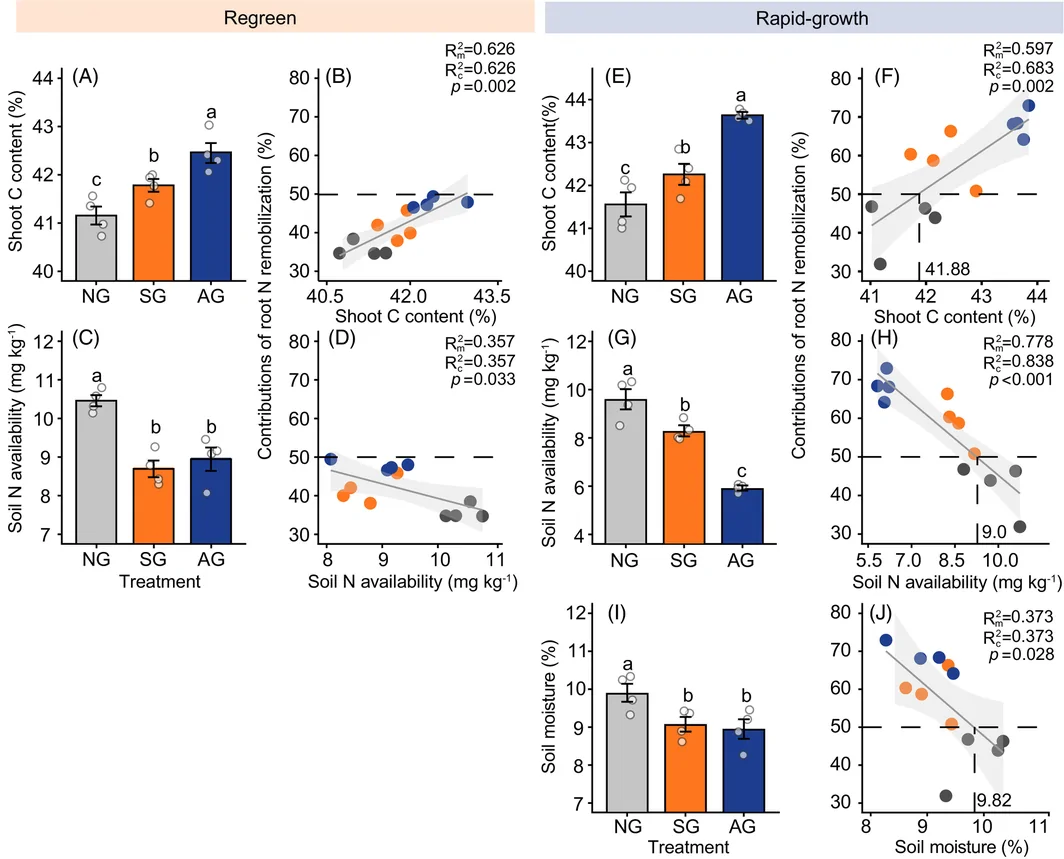
Changes in plant aboveground growth demand and soil N supply (soil N availability and moisture) indicators in two plant growth stages and across grazing treatments, and their relationships with the plant N use strategies (relative contribution of root N remobilization). (A, C, E, G, I) Changes in growth demand (shoot C content) and soil N supply (soil N availability and moisture) under three grazing treatments and during two plant growth stages. (B, D, F, H, J) Relationships of these indicators with the relative contribution of root N remobilization to aboveground N accumulation (Cre). NG, no‐grazing; SG, summer grazing; AG, autumn grazing. The column charts with error bars show mean ± SE; different letters indicate significant differences among grazing treatments (LSD test, *P* < 0.05, *n* = 12). The linear regressions were shown with gray‐shaded 95% confidence intervals, and a 50% line indicating the shift in the contribution of two N strategies. R^2^ and *P*‐values are derived from linear mixed models, with R^2^_m_ and R^2^_c_ representing the marginal and conditional R^2^, respectively.

## DISCUSSION

The trade‐off between root N remobilization and soil N uptake in plant aboveground N acquisition implies a dynamic in plant N use strategies between conservative and acquisitive strategies (Thornton et al., 2000). However, the dynamics of this trade‐off across seasons in grassland plants and the direction and magnitude of its responses to grazing are poorly understood. Our study provides empirical evidence that plant N use strategy leans toward acquisitive soil N uptake in the regreen stage but toward conservative N remobilization in the rapid‐growth stage, and that grazing induces a more conservative N use strategy. The variation in plant N use strategies across seasons or among grazing management is driven by the plant aboveground growth demand (sink) and the relative abundance of belowground N supply from soil N uptake and root N remobilization (source).

### Grazing leans the plant N use trade‐off toward conservative internal cycling

Our results show that plant aboveground N acquisition relies more on soil N uptake than root N remobilization in ungrazed grasslands across growth stages. However, this pattern changes in response to grazing disturbance. During the rapid‐growth stage, plants under grazing used more stored N remobilization than soil N uptake (Figures 1 and 2), a shift likely driven by grazing‐induced reductions in soil N availability, the primary factor controlling internal N cycling (Figure S1d, Figure 3H). Our findings confirm hypothesis I, showing that grazing shifts the plant N use trade‐off toward a more conservative N remobilization strategy. This aligns with previous observations and is most likely a consequence of plant adaptation to grazing‐induced declines in soil N availability (Li et al., 2022; Ru et al., 2024). The enhanced internal N cycling is a critical mechanism enabling rapid photosynthetic recovery after defoliation (Sun et al., 2018), as indicated by the high proportion of remobilized N in regrown shoots shortly after grazing (Dong et al., 2023; Li, Li, et al., 2025). The grazing‐enhanced photosynthetic activity is also indicated by the increased shoot C content in our studied grazing grassland (Figure S1e).

Our results also show that the mechanisms by which grazing affects the trade‐off between N remobilization and uptake differ in grasslands subject to summer or autumn grazing. The impact of summer grazing is achieved by mitigating soil N uptake, while that of autumn grazing is achieved by improving N remobilization. The suppression of summer grazing on soil N uptake is likely associated with its perturbation of plant growth and inhibition of root growth (Ourry et al., 1988; Thornton & Millard, 1996) (Figure 4B,C; Figure S2), while the enhancement of N remobilization by autumn grazing is associated with better plant growth, thus bigger aboveground N demand. In an N‐deficient environment, this demand cannot be met by the increased internal N cycling (Skinner et al., 1999).

The grazing induced shift in plant N use trade‐off toward N remobilization represents a critical physiological adjustment of plants under the cumulative impacts of grazing on plant physiological status and the surrounding resource environment. It is not merely a transient, pulse response to defoliation events. This sustained shift underscores the long‐term effects of grazing on plant nutrient economy and the ecosystem N cycle.

### Plant N use trade‐off shifts from acquisitive to conservative as the plant growth stage progresses

The trade‐off between plant root N remobilization and soil N uptake exists throughout the plant life cycle (Marmagne et al., 2022; Reich, 2014). Our result demonstrates that plants rely more on soil N uptake during the regreen stage, but more on internal N cycling during the rapid‐growth stage (Figure 1B,D). This shift happens mainly in grazed grassland, but not in ungrazed grassland (Figure 2). The significant interaction between grazing and plant growth stages on plant N use strategy (Table 1) confirms differences in the dynamics of the plant N use trade‐off between grazed and ungrazed grasslands. Our observed more acquisitive N use in the regreen stage rejects our hypothesis II and is contrary to the results obtained from several greenhouse studies showing that the herbaceous plants rely more on internal N cycling at the early growth stage (58–80%) (Bausenwein et al., 2001; Bausenwein & Millard, 2001). This contradiction may stem from a potentially broad N economics spectrum across herbaceous species, which has been observed in woody plants (Millard & Grelet, 2010; Rothstein & Zak, 2001). It is also possible that the actual responses of plant N use in natural habitats are regulated by complex factors, such as precipitation and N mineralization (Schimel & Bennett, 2004), which can be masked in greenhouse studies. Our results were obtained from an *in situ* experiment in a native grassland, in contrast to those from hydroponics or greenhouse experiments, and we propose that this discrepancy is not an artifact, but rather reflects a fundamental difference between the steady‐state resource conditions of controlled environments and the resource dynamics of natural ecosystems. In our native grassland, moisture is higher during the melting snow period, and available N is generally high during the regreen stage (Figure S1a,b). Under these conditions, the energetic and physiological costs of acquiring N from the soil are likely minimized. We therefore argue that, even for the modest N demand of initial shoot development, direct uptake from a rich soil represents a more efficient strategy than root N remobilization. Our *in situ* results thus provide a critical field‐based perspective, suggesting that plant N use strategies are highly plastic and responsive to the prevailing soil resource availability.

In contrast to our hypothesis II, our results show that plants rely more on root N remobilization than soil N uptake during the rapid‐growth stage, especially in grazed grassland (Figure 2). The grazing‐induced shift appears to be a classic response to a source‐sink imbalance, a mechanism crucial for understanding plant compensatory growth. Grazing stimulates rapid regrowth, creating a strong sink demand for N that outpaces the rate of soil N supply. This heightened internal demand is reflected in increased shoot C content (Figure 5E), likely indicating enhanced C assimilation to support regrowth. Concurrently, grazing pressure led to a decline in both soil N availability and moisture (Figure 5G,I), thereby increasing the costs and limitations of external N acquisition. This result aligns with that from previous studies (Jaeger & Monson, 1992; Millard & Grelet, 2010) that plants with vigorous growth potential rely more on conservative N remobilization when soil N uptake is insufficient. This reliance is often driven by the high cost of soil N uptake when availability is low (Dijkstra et al., 2018; Zhou, 2016). Our results from the *in situ* experiment support that the relative contributions of N remobilization versus uptake depend on the dynamic interplay between the size of the root N and soil N availability and their acquisition costs, as plant aboveground growth demand and soil N supply drive different components of total N flux.

### The shift in plant N use strategies is governed by plant growth demand and soil supply relations

The trade‐off in plant N use strategies between root N remobilization and soil N uptake is crucial for the resistance to grazing and subsequent regeneration of perennial plants (Kavanová & Gloser, 2005; Klimešová et al., 2018). Our results highlight the key roles played by plant growth N demand (sink) and soil N supply, rather than plant root N stock (source), in governing the trade‐off in plant aboveground N use strategies (Figure 3). Our results not only validate hypothesis III but also provide a mechanistic understanding of soil N supply versus root N remobilization. This result implies that previous understanding may have overestimated the importance of root N stock after defoliation (Ourry et al., 1994; Zhang et al., 2022). Plant roots play a dual role in acquiring external N and remobilizing stored N; however, no significant correlation is observed between root N stock and any flux of the two N use strategies and their trade‐off (Figure 3). The possible reason is that the amount of N remobilized is less affected by short‐term soil N supply. In addition, root N remobilization is not merely a passive process but proceeds in coordination with plant growth demand (Masclaux‐Daubresse et al., 2010; Thornton & Millard, 1996). This means that the root may merely be a transporter rather than a regulator of plant N acquisition. Our findings support the existence of threshold‐dependent regulation of plant N‐use trade‐offs by source‐sink traits and provide a preliminary estimate of the potential balance point between soil N uptake and internal N remobilization. This threshold is growth‐stage dependent, as plants robustly rely more on soil N uptake during the early growth stage. In contrast, plant N use trade‐off leans toward conservative internal cycling when source traits (N and water availability in soil) are below, or sink traits (shoot C content) are above the thresholds during rapid‐growth stage. Given that shifts in plant nutrient‐use strategies may follow nonlinear, abrupt, or asymptotic response patterns, the threshold derived from our linear models should be regarded as an approximate estimate rather than a precise ecological threshold. Moreover, whether these thresholds are broadly applicable or vary among regions remains unresolved, given the limited experimental scale and the focus on a restricted set of plant species. The universality of the findings for different plant species and in various ecosystems warrants further study.

## MATERIALS AND METHODS

### Study area

The study was conducted in a semiarid grassland in the research and demonstration farm of Inner Mongolia University, located in Xilinhot, Inner Mongolia, China (116°31′ E, 44°15′ N, 1146 m asl). The region experiences a semiarid, temperate continental climate, with a mean annual temperature of 4.2°C and a mean annual precipitation of 307 mm, 80% of which falls during the plant growing season from May to September (averaged over the period from 2017 to 2023). The experimental grassland is dominated by *Leymus chinensis* and *Stipa grandis*, with *Cleistogenes squarrosa* and *Carex korshinskyi* as common species (Table S1). The soil is a chestnut sandy loam.

### Experiment design of seasonal grazing

The grazing experimental grasslands, established in 2016, consist of 12 grassland plots of 2500 m^2^ (50 m × 50 m), which were used to arrange three grazing treatments of summer grazing (SG), autumn grazing (AG), and no grazing (NG, control), replicated in four blocks. Summer grazing includes three grazing rotations of six sheep in early June, July, and August, respectively. Grazing intensity was controlled at a moderate level by moving the sheep out of the plots when the mean plant residual height reached approximately 6 cm in each grazing rotation. Autumn grazing was conducted in mid‐September, and grazing intensity was maintained at the same level as summer grazing by setting 18 sheep in the plots to graze the same number of sheep‐grazing days (= number of sheep × number of grazing days). The no‐grazing plots were fenced to exclude sheep grazing.

### Field ^15^N isotope labelling and sampling

To ensure representativeness and homogeneity, we selected plant community patches where *Leymus chinensis* was predominant, accounting for approximately 85% of the total plant biomass on average. Plant N use processes (N remobilization versus uptake) in plant community patches dominated by *L. chinensis* were quantitatively evaluated by ^15^N isotope tracer in 2023. Measurements were done at four plant growth stages (regreen, rapid‐growth, peak biomass, and senescence) using 32 mesocosms of plant community patches in each experimental plot. For each measurement, eight mesocosms were established in representative and homogeneous *L. chinensis* patches within one quadrant of each experimental plot 30 days before isotope labelling, by embedding eight polyvinyl chloride (PVC) pipes (30 cm diameter and 30 cm depth) into the soil. To minimize variation arising from spatially heterogeneous livestock foraging, manual clipping was used with standardized defoliation intensity. In the summer grazing plots, direct livestock access to the mesocosms was prevented using 1‐m‐high cages. Vegetation was clipped to a residual height of 6 cm at the post‐regreen, rapid‐growth, and peak‐biomass stages, with all harvested biomass removed. No clipping was applied to the autumn grazing plots because grazing was conducted after the completion of plant sampling. Among the eight mesocosms used in each measurement in a plot, three paired mesocosms were used for plant sampling and the other two mesocosms for soil sampling. That is, plant above‐ and belowground tissues were sampled from three mesocosms (i.e., one mesocosm in each paired mesocosm for plant sampling) on the day when the remaining five mesocosms were labeled using the ^15^N isotope. The 51 mg K^15^NO_3_ tracer (99 atom% ^15^N) was applied to each of the five mesocosms, which have an average soil mass of ~30 kg. This dosage constituted approximately 5% of the total available soil N pool (5 mg kg^−1^), preventing fertilization artifacts. Each mesocosm received pulse‐labelling via uniform injection of 400 mL K^15^NO_3_ solution throughout the 0–30 cm soil profile. To ensure the spatially uniform labelling, we used a circular template with three concentric rings and 20 evenly distributed injection points. A hole with 25 cm depth was made with a steel needle at each point. The syringe was then inserted to the bottom of the hole, and 20 mL of solution was injected at a constant rate while the syringe was slowly withdrawn to the soil surface. Plant tissues were harvested from the three ^15^N‐labeled mesocosms as plant samples on the 14th day, while soil samples in the remaining two ^15^N‐labeled mesocosms were collected at six timings, that is, at 2 h (Day 0), 1st, 3rd, 6th, 10th, and 14th day after ^15^N labelling (Table S2). A soil core (3 cm in diameter and 30 cm in depth) was collected from each mesocosm at each sampling time and pooled into a single sample for each plot. The soil samples were sieved through a 2‐mm mesh and divided into dry and fresh samples. The fresh samples were extracted with 2 M KCl and analyzed for NH_4_^+^‐N and NO_3_^−^‐N concentrations using a continuous‐flow spectrophotometer (AA3, SEAL Analytical, Wrexham, UK). Soil moisture content was determined after oven‐drying aliquots at 105°C. The air‐dried samples were processed to determine total N using an elemental analyzer (*Vario PYRO Cube*, Elementar, Langenselbold, Germany). Plant samples were oven‐dried at 65°C for over 48 h and weighed. Dry plant materials were finely pulverized using a mixer mill (MM 400, RETSCH, Haan, Germany). The ^15^N isotope abundance of plant and soil samples was determined using an isotope ratio mass spectrometry (Isoprime100, Isoprime, Cheadle, England). Plant total C and N were also determined using the elemental analyzer. Soil temperature was obtained by the long‐term data monitoring system ZL6 (METER, Pullman, WA, USA).

### Calculation and statistics

The fluxes and contributions of plant root N remobilization and soil N uptake were assessed across different plant growth stages and grazing treatments. The relationships of plant N remobilization with plant aboveground growth demand, soil N supply, and root N stock were analyzed. Plant N demand for aboveground growth represents sink characteristics, whereas soil N supply and root N stock constitute source characteristics. The change in plant aboveground N pool (AGN) signifies the direction of plant internal N cycling; a decrease implies a resorption of aboveground N, whereas an increase implies an absorption of N from soil or root stock. Changes in plant AGN were calculated for each 14‐day experimental period across four plant growth stages, and the results showed that N remobilization occurred at the regreen and rapid‐growth stages, while N resorption occurred at the peak biomass and senescence stages, particularly in grasslands under summer grazing (Figures S3 and S4). Thus, the fluxes and contributions of root N remobilization and soil N uptake to plant AGN accumulation were calculated only for regreen and rapid‐growth stages.

Contributions of plant root N remobilization (Cre) and soil N uptake (Cup) were calculated based on the equations from (Wang et al., 2021) (Equations 1 and 2), and the deductions of these equations are in the Supporting Information.

(1) $$\mathrm{Cre}=\frac{\;\delta_{s}-\delta_{p}\;}{\delta_{s}-\delta_{r}}\times 100\%$$

(2) $$\mathrm{Cup}=\frac{\;\delta_{p}-\delta_{r}\;}{\delta_{s}-\delta_{r}}\times 100\%$$

where $\delta_{r}$ and $\delta_{p}$ are ^15^N isotope abundance in plant roots before labelling ($\delta_{r}$ ≈ 0.367%) and plant aboveground tissues at 14 days after labelling. $\delta_{s}$ is the time‐weighted mean ^15^N isotope abundance of soil N availability during the experimental period (14 days). The measured soil total N (TN) and available N content at six timings (i = 0, 1, 3, 6, 10, and 14 days) were used to calculate ^15^N isotope abundance in soil available N (Equation 3).

(3) $$\delta_{i}=\frac{\;\left(\delta_{i}-\delta_{0}\right)\times \mathrm{TN}+\delta_{0}\times \text{AN}\;}{\text{AN}}$$

where $\delta_{i}$ and $\delta_{0}$ are respectively measured ^15^N isotope abundance in soil at the ith sampling after labelling and natural ^15^N isotope abundance in soil. Thus, the ^15^N isotope abundance of available N was estimated as the time‐weighted mean abundance of its relative duration (Equation 4).

(4) $$\delta_{s}=\sum_{1}^{5}\frac{\;\delta_{i}+\delta_{i+1}\;}{2}\times \frac{\;D_{i}\;}{14}$$

where $D_{i}$ is the duration in days between the ith and i + 1th samplings.

The fluxes of root N remobilization (Fre) and soil N uptake (Fup) were the product of their contribution rates and increased plant AGN pool (Equations 5 and 6).

(5) Fre=Cre×ΔAGN

(6) Fup=Cup×ΔAGN

where ΔAGN is the increase of plant AGN pool during the labelling experimental period (14 days).

All statistical analyses were conducted in R (version 4.4.2). To minimize measurement error, the technical replicates within each grassland plot were averaged to yield a single value. This resulted in a final dataset of 24 independent data points, each representing a single experimental plot, for statistical analyses.

The effects of plant growth stage, grazing treatments, and their interactions on the fluxes and contributions of two plant N pathways were analyzed using linear mixed models (LMMs) in R with the ‘*lme4*’ package (Bates et al., 2014). In each model, grazing treatment, growth stage, and their interaction were included as fixed effects, and block was included as a random intercept. Fixed effect significance was assessed using Type III ANOVA tables with Satterthwaite's degrees of freedom approximation from the “*lmerTest”* package (Kuznetsova et al., 2017). Post hoc pairwise comparisons used estimated marginal means (EMMs) with a LSD adjustment for multiple comparisons, implemented via the “*emmeans”* package.

Hierarchical partitioning was employed to determine the individual contribution of each predictor in the explained variance of the full model in driving plant N use strategies, using the “*glmm.hp*” package (Lai et al., 2022). Predictors representing plant growth demand differed between the relative‐contribution and flux models. We used ΔAGN as a predictor for Cre, while ΔAGB as a predictor for Fre and Fup, because ΔAGN was included in the calculation of both fluxes. Shoot C content was also included as a predictor of aboveground growth demand. Soil N supply (soil N availability, soil moisture, and root N stock (belowground biomass and N pool)) was included in the models. All predictors were standardized using global min‐max normalization before model fitting. Relationships between plant N use strategies and key environmental or plant factors were visualized, and model fits (e.g., R^2^) were extracted from the mixed‐effects models described above. Where a significant relationship was observed, its intersection with the reference line was used to estimate a potential threshold in the plant N‐use trade‐off. This model predicted intersection denotes the balance point at which the relative dominance of soil N uptake and root N remobilization changes. All figures were generated using the “*ggplot2”* package (Wickham, 2016).

## AUTHOR CONTRIBUTIONS

FYL and TZ conceived and designed the study. LW, KL, ZM, HW, YL, YW, PX, and ZJ did the field experiment. LW analyzed data and led the manuscript writing with contributions from TZ and FYL. All authors contributed to and approved the manuscript.

## CONFLICT OF INTEREST

The authors declare that they have no known competing financial interests or personal relationships that could have appeared to influence the work reported in this paper.

## Supporting information


**Figure S1.** Soil and plant indicators in plant regreen and rapid‐growth stages (a–c) or under summer grazing (SG), autumn grazing (AG), or no‐grazing (NG) (d–e) in a semi‐arid grassland. The column chart with error bars shows mean ± SE. Different lowercase letters indicate significant differences between plant growth stages or among grazing treatments (d, e) (*n* = 24, *P* < 0.05).
**Figure S2.** Changes in plant aboveground N pool (AGN) under summer grazing (SG) or autumn grazing (AG). The column chart with error bars shows mean ± SE. Different lowercase letters indicate significant differences among grazing treatments (*n* = 16, *P* < 0.05).
**Figure S3.** Changes in the plant aboveground nitrogen pool (AGN) in the four plant growth stages in grasslands subject to summer (SG), autumn (AG), or no‐grazing (NG). The column chart with error bars shows mean ± SE (*n* = 12).
**Figure S4.** Plant aboveground N content at the time of ^15^N labelling (start) and 14 days later (end) at the plant peak‐biomass stage in the grassland subject to summer (SG), autumn (AG), or no‐grazing (NG). The column chart with error bars shows mean ± SE. Significant differences between the start and end points are marked (*P < 0.05, *n* = 24).
**Figure S5.** Percentage contribution of *Leymus chinensis* to total aboveground biomass within community patches in the mesocosms in grazing and no‐grazing grassland plots (left), and a photograph of a mesocosm (right).
**Table S1.** Plant species composition of the grasslands under study.
**Table S2.** Sampling scheme of the eight mesocosms in each measurement in a plot (three mesocosms for plant sampling at the start (Plant_start), another three for plant sampling at the end of measurement (Plant_end) on the 14th day after the ^15^N labelling, and the remaining two for soil sampling during the 14‐day period). N, number of mesocosms; ✔ indicates a sampling.
**Table S3.** Results (*F*‐values, degrees of freedom (df), and *P*‐values) of a linear mixed model on the effects of grazing treatments on the contribution of root N remobilization to plant aboveground N accumulation (C_re_) in each plant growth stage. Twelve observations (*n* = 12) were made for 3 grazing treatments × 4 replicates at each growth stage (data for Figure 2).

## Data Availability

The data that support the findings of this study are available upon request from the corresponding author. The data are not publicly available due to privacy or ethical restrictions.
