# Supplementary material for: Grazing reshapes plant nitrogen use trade‐off between soil uptake and internal remobilization
Source: Plant J. 2026 Oct 12;128(1):e71168. doi: 10.1111/tpj.71168 (PMC13656187; doi:10.1111/tpj.71168)
Supplement: Supplementary file 1 [file TPJ-128-0-s001.docx]

**Grazing reshapes plant nitrogen use trade-off between soil uptake and internal remobilization.**

Lin Wu^a,b^, Kexin Li^a^, Ziyuan Ma^a^, Hao Wang^a^, Yanlong Li^a,d^, Yadong Wang^a,b^, Pengfei Xing^a,b^, Zhicheng Jin^a^, Tongrui Zhang^c,^*, Frank Yonghong Li^a,b,^*

^a^ Ministry of Education Key Laboratory of Ecology and Resource Use of the Mongolian Plateau & Inner Mongolia Key Laboratory of Grassland Ecology, School of Ecology and Environment, Inner Mongolia University, 235 West University Road, Hohhot 010021, China

^b^ Collaborative Innovation Center for Grassland Ecological Security, Ministry of Education of China, Inner Mongolia University, Hohhot, 010021, China

^c^ Institute of Grassland Research, Chinese Academy of Agricultural Sciences, Hohhot 010010, China

^d^ Observation and Research Station for Steppe Ecosystems of the Ministry of Education of China, Xilingol, Inner Mongolia, China.

***** Author for correspondence:

Tongrui Zhang: zh4ngtongrui@163.com

Frank Yonghong Li: lifyhong@126.com

**Figure S1.** Soil and plant indicators in plant regreen and rapid-growth stages (a-c) or under summer grazing (SG), autumn grazing (AG), or no-grazing (NG) (d-e) in a semiarid grassland. The column chart with error bars shows mean ± SE. Different lowercase letters indicate significant differences between plant growth stages or among grazing treatments (d, e) (n=24, *P* < 0.05).

**Figure S2.** Changes in plant aboveground N pool (AGN) under summer grazing (SG) or autumn grazing (AG). The column chart with error bars shows mean ± SE. Different lowercase letters indicate significant differences among grazing treatments (n=16, *P* < 0.05).

**Figure S3.** Changes in the plant aboveground nitrogen pool (AGN) in the four plant growth stages in grasslands subject to summer (SG), autumn (AG), or no- grazing (NG). The column chart with error bars shows mean ± SE (n=12).

**Figure S4.** Plant aboveground N content at the time of ^15^N labelling (start) and 14 days later (end) at the plant peak-biomass stage in the grassland subject to summer (SG), autumn (AG), or no- grazing (NG). The column chart with error bars shows mean ± SE. Significant differences between the start and end points are marked. (* *p* < 0.05, n=24).

**Figure S5.** Percentage contribution of *Leymus chinensis* to total aboveground biomass within community patches in the mesocosms in grazing and no-grazing grassland plots (left), and a photo of a mesocosm (right).

**Table S1.** Plant species composition of the grasslands under study

| Number | Species name | Family | Functional group |
| --- | --- | --- | --- |
| 1 | *Leymus chinensis* | Gramineae | PG |
| 2 | *Stipa grandis* | Gramineae | PG |
| 3 | *Cleistogenes squarrosa* | Gramineae | PG |
| 4 | *Carex korshinskyi* | Cyperaceae | PG |
| 5 | *Thalictrum petaloideum* | Ranunculaceae | PF |
| 6 | *Allium ramosum* | Amary llidaceae | PF |
| 7 | *Astragalus galactites* | Leguminaceae | PF |
| 8 | *Potentilla acaulis* | Rosaceae | PF |
| 9 | *Artemisia frigida* | Compositae | PF |
| 10 | *Artemisia scoparia* | Compositae | A/B |
| 11 | *Setaria viridis* | Gramineae | A/B |
| 12 | *Lappula intermedia* | Boraginaceae | A/B |
| 13 | *Chenopodium aristatum* | Chenopodiaceae | A/B |
| 14 | *Chenopodium album* | Chenopodiaceae | A/B |
| 15 | *Salsola collina* | Chenopodiaceae | A/B |
| 16 | *Silene jenisseensis* | Caryophyllaceae | A/B |

PG-perennial grasses, PF-perennial forbs, A/B-annuals/biennials.

**Table S2.** Sampling scheme of the eight mesocosms in each measurement in a plot (3 mesocosms for plant sampling at the start (Plant_start), another 3 for plant sampling at the end of measurement (Plant_end) on the 14^th^ day after the ^15^N labelling, and the remaining 2 for soil sampling during the 14-day period). N, number of mesocosms; ✔ indicates a sampling.

|  | |  | Sampling time | | | | | |
| --- | --- | --- | --- | --- | --- | --- | --- | --- |
| Group | N | Day 0 | Day 0 (2h) | Day 1 | Day 3 | Day 6 | Day 10 | Day 14 |
| Plant_start | 3 | ✔ |  |  |  |  |  |  |
| Plant_end | 3 | ^15^N Labelling |  |  |  |  |  | ✔ |
| Soil | 2 | ^15^N Labelling | ✔ | ✔ | ✔ | ✔ | ✔ | ✔ |

**Table S3.** Results (*F*-values, degrees of freedom (df), and *P*-values) of a linear mixed model on the effects of grazing treatments on the contribution of root N remobilization to plant aboveground N accumulation (C_re_) in each plant growth stage. Twelve observations (n=12) were made for 3 grazing treatments * 4 replicates at each growth stage. (data for Figure 2).

| Stages |  | Grazing treatments | | |
| --- | --- | --- | --- | --- |
|  | n | df | *F* | *P* |
| Regreen | 12 | 2 | 27.67 | <0.001 |
| Rapid-growth | 12 | 2 | 44.32 | <0.001 |

**The formula used in quantifying the contribution of plant root nitrogen (N) remobilization to aboveground N acquisition (C_re_) during the plant growth stages using the ¹⁵N isotope dilution technique.**

At the beginning of each experimental period, corresponding to the regreening and rapid-growth stages, a K^15^NO_3_ solution enriched to 99 atom% ^15^N was applied to the soil within each experimental microcosm. As the N accumulated in the aboveground tissues originates from two sources, the ^15^N labelled soil N and the remobilized root N, the resulting ^15^N abundance in the plant aboveground tissues is lower than that in the soil. The difference in ^15^N abundance between the plant tissues and the soil allows for the calculation of the contribution of remobilized belowground N reserves to the N acquired for aboveground growth during the growth stages.

Let:

- a = Quantity of N remobilized to the aboveground N pool from belowground storage during the growth stages.
- b = Quantity of N uptake from the soil and translocated to the aboveground N pool during the growth stages.

Therefore, the total aboveground N pool accumulation at the growth stages is (a + b).

Let:

- δr = ^15^N abundance (%) in the belowground storage tissues before the growth stages (and before tracer application).
- δp = ^15^N abundance (%) in the aboveground tissues at the end of the growth stages (and after tracer application).
- δs = ^15^N abundance (%) in the plant-available soil N pool during the growth stages (and after tracer application).

Therefore, the total amount of ^15^N accumulated in the aboveground N pool during the growth stages is the sum of the ^15^N remobilized from root storage and ^15^N newly root uptake from the soil (Eqn. 1). Then, by derivation, we obtain the formula for the contribution of the N remobilization to total plant aboveground N acquisition (Cre).

$$\delta_{r}\times a+\delta_{s}\times b=\delta_{P}\times\left( a+b \right) Eqn.1$$

$$b= \frac{{\delta_{r}-\delta}_{p}}{\delta_{p}-\delta_{s}} \times a Eqn.2$$

$$Cre= \frac{a}{a+b} \times100\%=\frac{a}{a+\frac{{\delta_{r}-\delta}_{p}}{\delta_{p}-\delta_{s}}\times a} \times100\% Eqn.3$$

$$Cre= \frac{{\delta_{s}-\delta}_{p}}{\delta_{s}-\delta_{r}} \times100\% Eqn.4$$
